# Effect of a cod protein hydrolysate on postprandial glucose metabolism in healthy subjects: a double-blind cross-over trial

**DOI:** 10.1017/jns.2018.23

**Published:** 2018-11-28

**Authors:** Hanna Fjeldheim Dale, Caroline Jensen, Trygve Hausken, Einar Lied, Jan Gunnar Hatlebakk, Ingeborg Brønstad, Dag Arne Lihaug Hoff, Gülen Arslan Lied

**Affiliations:** 1 Department of Clinical Medicine, Centre for Nutrition, University of Bergen, Bergen, Norway; 2 Division of Gastroenterology, Department of Medicine, Haukeland University Hospital, Bergen, Norway; 3 National Centre of Functional Gastrointestinal Disorders, Haukeland University Hospital, Bergen, Norway; 4 Firmenich Bjørge Biomarin AS, Ellingsøy, Ålesund, Norway; 5 Department of Clinical Medicine, University of Bergen, Bergen, Norway; 6 National Centre for Ultrasound in Gastroenterology, Haukeland University Hospital, Bergen, Norway; 7 Division of Gastroenterology, Department of Medicine, Ålesund Hospital, Møre & Romsdal Hospital Trust, Ålesund, Norway; 8 Department of Clinical and Molecular Medicine, Faculty of Medicine and Health Sciences, Norwegian University of Science and Technology, Trondheim, Norway

**Keywords:** Marine protein hydrolysate, Fish protein, Marine peptides, Glucose metabolism, BCAA, branched-chain amino acids, GLP-1, glucagon-like peptide 1, MPH, marine protein hydrolysate, T2DM, type 2 diabetes mellitus

## Abstract

The increased prevalence of lifestyle diseases, such as the metabolic syndrome and type 2 diabetes mellitus (T2DM), calls for more knowledge on dietary treatments targeting the specific metabolic pathways involved in these conditions. Several studies have shown a protein preload before a meal to be effective in lowering the postprandial glycaemic response in healthy individuals and patients with T2DM. The aim of the present study was to assess the effect of a marine protein hydrolysate (MPH) from Atlantic cod (*Gadus morhua*) on postprandial glucose metabolism in healthy, middle-aged to elderly subjects. This double-blind cross-over trial (*n* 41) included two study days with 4–7 d wash-out in between. The intervention consisted of 20 mg of MPH (or casein as control) per kg body weight given before a breakfast meal. The primary outcome was postprandial response in glucose metabolism, measured by samples of serum glucose, insulin and plasma glucagon-like peptide 1 (GLP-1) in 20 min intervals for 180 min. In a mixed-model regression analysis, no differences were observed between MPH and control for postprandial glucose concentration (mean difference: −0·04 (95 % CI –0·17, 0·09) mmol/l; *P* = 0·573) or GLP-1 concentration (mean difference between geometric means: 1·02 (95 % CI 0·99, 1·06) pmol/l; *P* = 0·250). The postprandial insulin concentration was significantly lower after MPH compared with control (mean difference between geometric means: 1·067 (95 % CI 1·01, 1·13) mIU/l; *P* = 0·032). Our findings demonstrate that a single dose of MPH before a breakfast meal reduces postprandial insulin secretion, without affecting blood glucose response or GLP-1 levels, in healthy individuals. Further studies with repeated dosing and in target groups with abnormal glucose control are warranted.

The proportion of the population with health problems related to overweight and obesity is constantly increasing worldwide, and this constitutes a great risk factor for several lifestyle diseases such as insulin resistance, the metabolic syndrome and type 2 diabetes mellitus (T2DM)^(^^1^^)^. The ability of the body to control postprandial glucose metabolism is decisive for health. Several dietary treatments for the prevention of postprandial hyperglycaemia in both diabetic and non-diabetic individuals have been suggested, but the necessary lifestyle and diet changes are challenging, and continue to lack adherence^(^^2^^)^. There is a need for more knowledge on dietary treatments targeting the specific metabolic pathways involved in overweight, obesity, the metabolic syndrome and T2DM.

Diets relatively high in protein (18–30 % energy) have been shown to be effective in the management of obesity due to suppression of appetite^(^^3^^)^, and are further suggested to reduce postprandial blood glucose in both healthy individuals and patients with impaired glucose metabolism^(^^4^^)^. Several trend diets have over the last decades included high-protein diets to reduce weight and suppress insulin response^(^^5^^,^^6^^)^, but the long-term effects of high-protein diets are unknown^(^^7^^,^^8^^)^. Furthermore, several studies have shown a protein preload before a meal to be effective in lowering the postprandial glycaemic response both in T2DM patients and healthy subjects^(^^9^^–^^13^^)^.

Due to limited access to high-quality protein in the world, it is neither sustainable nor possible for the world's population to increase the proportion of protein in the diet. Thus, the potential benefit of altering the source and quality of protein, rather than increasing the amount, is of great interest. Marine resources in excess should be evaluated as a possible high-quality protein source for human consumption^(^^14^^)^.

Previous studies in rats and human subjects have shown that the intake of both fish proteins and marine protein hydrolysates (MPH), even in low doses, has a desirable effect on insulin sensitivity and postprandial glucose^(^^7^^,^^15^^–^^19^^)^, lipids in serum and adipose tissue, bile acids, fatty acid composition and growth, and possibly has antihypertensive and immune-modulating effects^(^^14^^,^^19^^–^^23^^)^. It is indicated that MPH may contain marine bioactive compounds with potentially important biological effects in humans, beyond the known effect of protein as a source of amino acids^(^^24^^,^^25^^)^. The use of MPH as a dietary supplement with similar or better health benefits than a regular fish meal could be both cost-effective, environmentally friendly and sustainable. A low dose of MPH is presumed to be effective due to the content of bioactive peptides not equally present in other protein sources.

Thus, the present study was designed to assess the effect of a single, low dose of MPH before a meal on postprandial glucose metabolism in healthy, middle-aged to elderly subjects.

## Subjects and methods

### Trial design

The study was a double-blind cross-over trial, including two different study days, with a 4–7 d wash-out period in between. The intervention implemented 20 mg of MPH per kg body weight (test material) or control (casein). MPH or casein powder (identical, both flavoured with lemon) was mixed with water and taken before a standardised breakfast meal, in randomised order. The primary outcome was postprandial response in glucose metabolism, measured by venous samples of serum glucose and insulin, and plasma glucagon-like peptide 1 (GLP-1). The secondary outcome was adverse events measured by symptom questionnaires.

This study was conducted according to the guidelines laid down in the Declaration of Helsinki and all procedures involving human subjects were approved by the Regional Committees for Medical and Health Research Ethics of Central Norway (2017/1794). Written informed consent was obtained from all subjects. The trial was registered at clinicaltrials.gov as NCT03669796.

### Participants

**Fig. 1.**
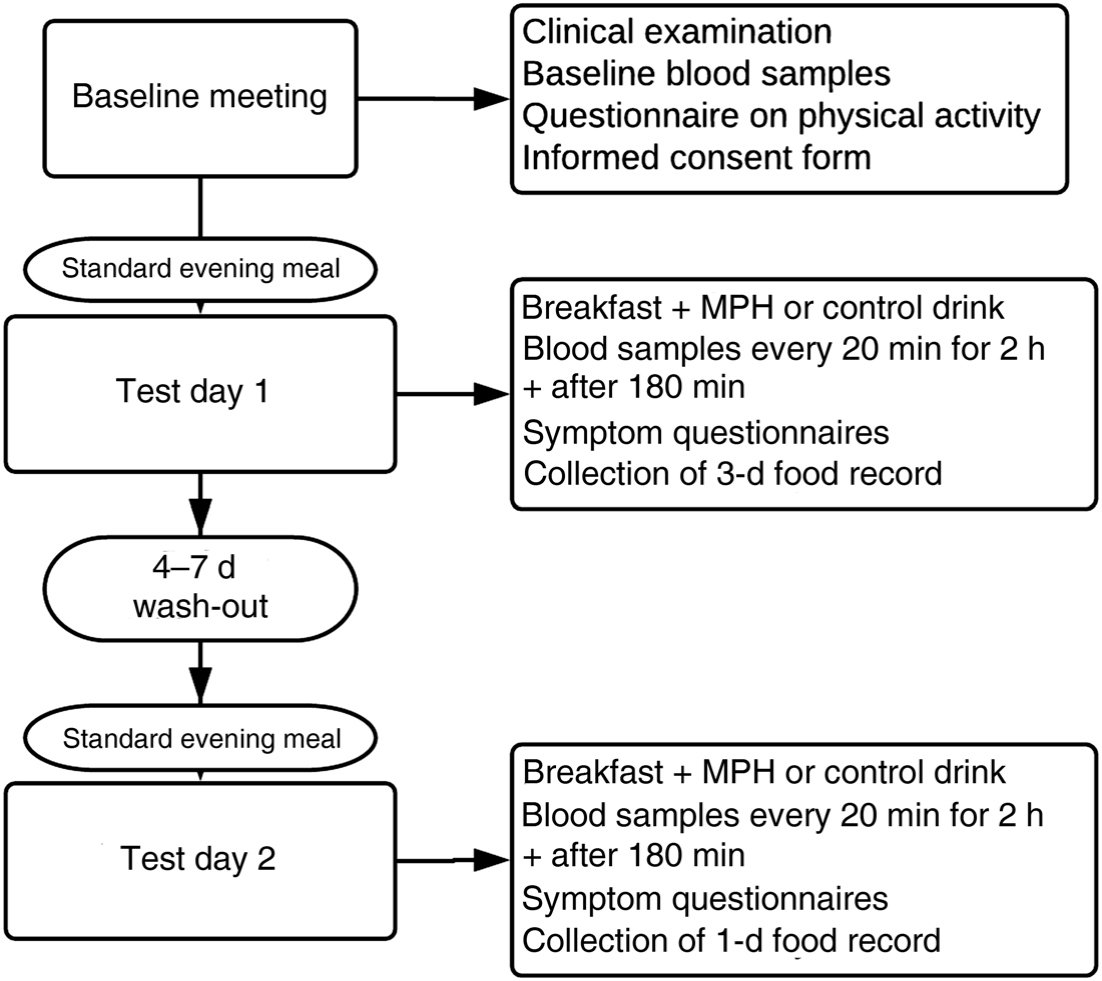
Flowchart depicting the inclusion process for the study evaluating the effect of a marine protein hydrolysate (MPH) from Atlantic cod (*Gadus morhua*) on postprandial glucose metabolism in healthy individuals aged 40–65 years. Participants were recruited through advertisements on the Internet and posters at Haukeland University Hospital and Ålesund Hospital between October 2017 and February 2018.

Participants were recruited through advertisements on the Internet and posters at Haukeland University Hospital and Ålesund Hospital between October 2017 and February 2018. Potential participants were interviewed for general eligibility and compliance with inclusion and exclusion criteria, and suitable candidates were invited for a further screening visit. A total of forty-one healthy and active individuals between 41 and 64 years old were included in the study (male, *n* 15; female, *n* 26). The inclusion process is depicted in Fig. 1.

Inclusion criteria were aged 40–65 years old and BMI 20–30 kg/m^2^. Exclusion criteria were fish allergy, pharmacologically treated diabetes mellitus, elevated blood pressure, chronic diseases (that might affect the evaluation of the study endpoints) and acute infections. The participants were instructed not to take any nutritional supplements containing *n*-3 fatty acids for 1 week before the study start, and while participating in the study.

### Study protocol

**Fig. 2.**
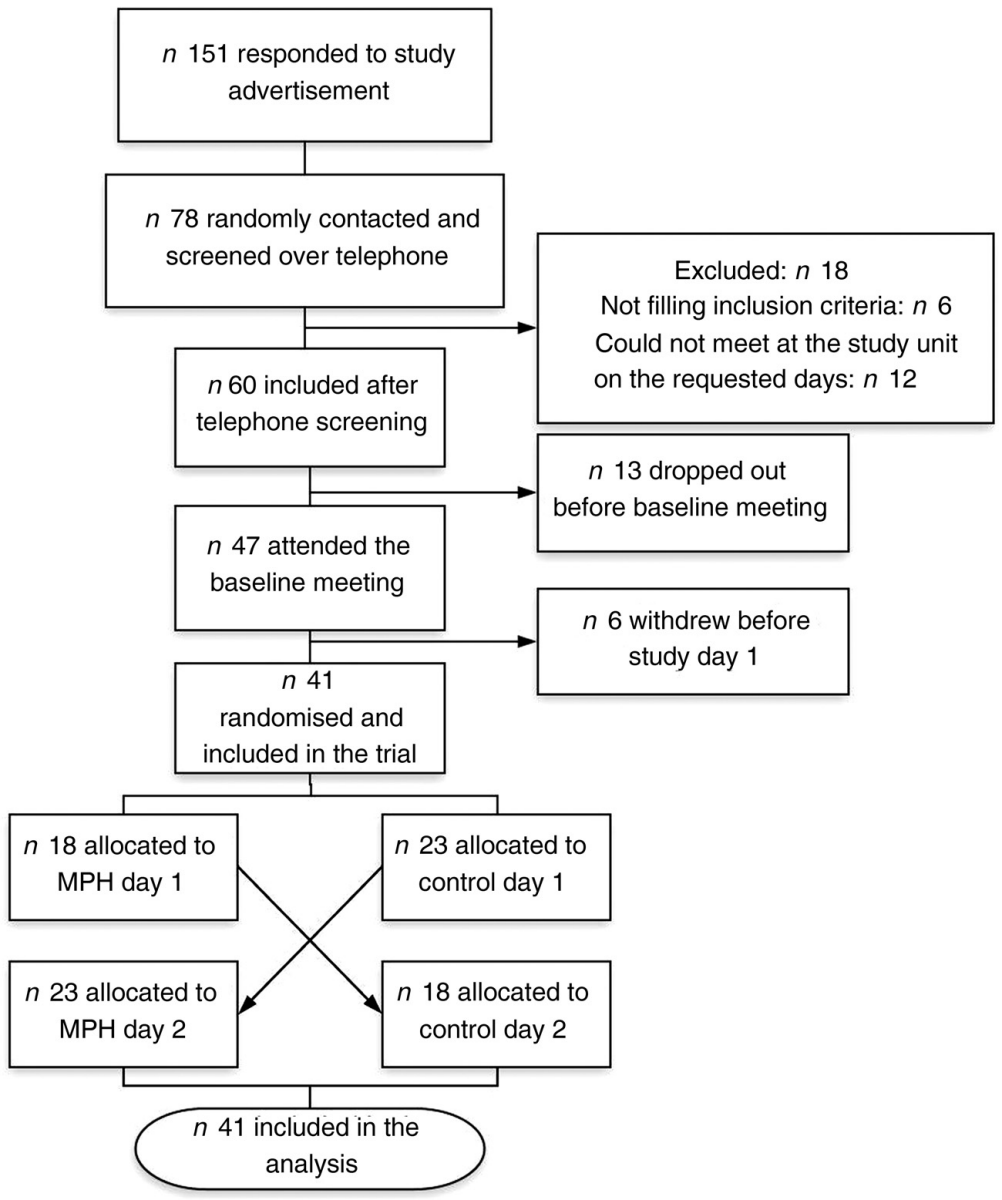
Study protocol for the evaluation of the effect of a marine protein hydrolysate (MPH) from Atlantic cod (*Gadus morhua*) on postprandial glucose metabolism. We included forty-one healthy subjects (age range 40–64 years).

The participants came to the research units on two different occasions, with a 4–7 d wash-out period (Fig. 2). A clinical examination by a physician, baseline biochemistry and measures of height, weight and blood pressure were done before inclusion. The level of physical activity was assessed, and participants were instructed not to change the level of physical activity or diet composition during the study period.

A 3-d and 1-d prospective dietary record was filled out prior to study days 1 and 2, respectively. On the day preceding each study day, the participants were provided with a standardised evening meal (oatmeal, rice or barley porridge) instructed to be eaten before 20.30 hours, followed by fasting until the next morning.

On study days, the participants came to the research units between 08.00 and 09.00 hours. After blood samples, they were served a drink with MPH or control, before a breakfast meal was given. The first post-meal sample (0 min sample) was taken 15 min after the breakfast was served.

The standardised breakfast meal consisted of two slices of semi-coarse bread (50 % whole wheat, 80 g bread), 10 g margarine, 20 g strawberry jam and 20 g white cheese, providing a total of 355 kcal (1485 kJ), 41 g carbohydrate, 12·5 g protein and 15 g fat. The drink provided on average 35·9 g carbohydrate and 145 kcal (607 kJ). Thus, including the drink, the breakfast provided in total 500 kcal (2092 kJ) and 77 g carbohydrate. The amount of energy and carbohydrates in the breakfast was calculated to induce an adequate blood glucose response. No coffee or tea was served, but water *ad libitum*.

The participants spent 4 h at the research units to allow for repeated sampling of blood, at 20 min intervals until 180 min, and monitoring of blood pressure.

### Assessments

Assessment of the participants’ medical history, and measurement of biochemical variables and safety parameters were conducted at baseline.

During the study days, serum glucose and serum insulin were measured at baseline and every 20 min for 2 h (120 min), with a final sample at 180 min. GLP-1 was measured at baseline, time 0, 20, 40, 80 and 180 min. Blood pressure was measured at baseline, after 40 min and after 180 min, as a safety parameter.

Two questionnaires evaluating the participants’ self-experienced symptoms were implemented to identify possible adverse events during each study day. A visual analogue scale was filled out six times during the study day, and a questionnaire validated for the evaluation of gastrointestinal symptoms (Kane) was filled out at baseline and at the end of each study day^(^^26^^)^.

### Estimation of nutritional intake

Calculations of energy and macronutrient intake were performed using *Kostholdsplanleggeren* (Norwegian Food Safety Authority and The Norwegian Directorate of Health, Oslo, Norway)^(^^27^^)^. The dietary records were used to evaluate the composition of the baseline diet, to map the participants’ regular meal pattern and to compare the days prior to each study day according to energy intake.

### Test materials

The MPH and casein powder were delivered from the manufacturer (Firmenich Bjørge Biomarin AS) in neutral bottles coded with participant number and study day. The bottles were coded by a person not involved in the implementation of the study and randomised according to a randomisation list. Both study participants and all persons involved in study conduction and analysis were blinded. The powder contained 4 % protein (MPH raw material or casein) and 96 % carbohydrate (maltodextrin). It was flavoured with lemon, but otherwise neutral. It was not possible to identify the active ingredient from the control, according to flavour or appearance. Each participant was given 20 mg/kg body weight of MPH or control. The drinks were made isonitrogenous, and equal amounts of N in the form of casein were added to the control drink. This was done to avoid any bias due to difference in N content between the MPH drink and the control drink. The amount of protein (N x 6·25) in both drinks was on average 1·6 g, constituting only a small fraction of the total protein content of the standardised breakfast meal. Casein was chosen as the control as it has previously shown to not affect blood glucose or insulin sensitivity when compared with proteins from cod and soya^(^^28^^)^.

The MPH powder was made by Firmenich Bjørge Biomarin AS by hydrolysing fish meat of Atlantic cod (*Gadus morhua*) with Protamex® (Novozymes AS) followed by spray drying of the soluble part of the enzyme digest. The MPH raw material contained approximately 89 % protein by weight, <0·2 % fat, 0 % carbohydrate, <3·0 % water, 10 % ash, 0·1 % NaCl, 1·7 % Na and 0·07 % chloride. Free amino acids accounted for 4·77 % of total amino acids in the MPH, and the essential amino acids:non-essential amino acids ratio was 0·70. Analysis of the molecular weight distribution (Table 1) shows that about 90 % of the peptides in the fish protein hydrolysate have a molecular weight of 2000 Da or less (eighteen amino acids or fewer), about 75 % of 1000 Da or less (ten amino acids or fewer) while about 55 % have a molecular weight of 500 Da or less (five amino acids or fewer). Approximately 25 to 30 % of the peptides have a molecular weight less than 200 Da, which represents small dipeptides and free amino acids.

**Table 1.** Molecular weight distribution in the dry and solubilised marine protein hydrolysate produced from meat of Atlantic cod (*Gadus morhua*)

Molecular weight (Da)	Amino acid moieties	g/100 g soluble peptides	g/100 g in the spray-dried powder
>10 000	–	<0·1	<0·1
10 000–8000	88–71	0·1	0·1
8000–6000	70–53	0·6	0·5
6000–4000	52–36	2·1	1·9
4000–2000	35–18	7·2	6·3
2000–1000	17–10	14·8	13·0
1000–500	9–5	21·0	18·5
500–200	4–2	27·0	23·8
<200	≤2	27·2	24·0

The casein contained approximately 88 % protein. The amino acid composition of MPH and casein used as control is presented in Table 2 (data obtained from Firmenich Bjørge Biomarin AS).

**Table 2.** Amino acid and taurine composition of the marine protein hydrolysate (MPH) from Atlantic cod (*Gadus morhua*) and the casein control used in the present study

Amino acid	Total amino acids (mg/g)	
	MPH	Casein (control)
Alanine	47·8	28·9
Arginine	51·1	32·0
Aspartic acid	73·3	70·8
Asparagine	0·38	N/A
Glutamic acid	125·0	213·5
Glutamine	0·78	N/A
Glycine	50·9	15·9
Histidine	13·5	24·4
Hydroxyproline	1·0	N/A
Isoleucine*	30·1	46·1
Leucine*	60·3	86·1
Lysine*	71·3	76·3
Methionine	22·1	25·8
Phenylalanine	23·2	47·2
Proline	29·7	95·3
Serine	36·0	50·3
Taurine	6·6	N/A
Threonine	30·9	38·8
Tryptophan	6·0	11·0
Tyrosine	22·7	47·8
Valine	36·9	59·4

N/A, not available.

* Branched-chained amino acids.

The MPH was analysed at the Allergy Laboratory (Haukeland University Hospital, Bergen, Norway) for allergenicity of the hydrolysate. Direct ELISA showed insignificant reactivity of specific IgG and IgE to the hydrolysate in comparison with the reactivity against cod allergen. The allergenicity of the hydrolysate was so low that it was considered unsignificant.

### Analysis of blood samples

Baseline biochemistry was analysed according to standard accredited methods at the Laboratory for Clinical Biochemistry, Haukeland University Hospital (Bergen, Norway) and the Department of Medical Biochemistry, Ålesund Hospital (Ålesund, Norway).

Glucose and insulin were measured in serum according to standard accredited methods at the Laboratory for Clinical Biochemistry, Haukeland University Hospital (Bergen, Norway). Serum was obtained by centrifugation of full blood at 2000 ***g*** at room temperature (20°C) for 10 min after 30–60 min of coagulation, using serum separator cloth activator tubes. Samples were aliquoted and stored frozen at −80°C prior to analyses.

Plasma for GLP-1 determination was obtained by centrifugation of EDTA full blood at 1800 ***g*** at −4°C for 10 min within 20 min after blood sampling. To EDTA blood sampling tubes were added 10 µl dipeptidyl peptidase-4 inhibitor (DPP4-010; DRG Diagnostics) per ml EDTA blood prior to sampling. GLP-1 plasma was aliquoted and stored frozen at −80°C prior to analysis. The GLP-1 analyses were performed using an ELISA kit from IBL International GmbH (GLP-1 (7–36) active ELISA, reference RE53121).

### Statistical analysis

Statistical analysis was performed using SPSS software (IBM SPSS Statistics 24) and GraphPad Prism version 7.0 (GraphPad Software, Inc.). The Shapiro–Wilk test was used to assess normal distribution. Mixed-model regression analysis was conducted to evaluate the difference between MPH and control. Non-normally distributed data were log-transformed before analysis (insulin and GLP-1) and are presented as log mean and back-transformed values. Paired *t* tests were used to evaluate differences in nutrient intake between study days. Two-way ANOVA with repeated measures was used to evaluate differences between each time point. Graphical work was conducted in GraphPad Prism. *P* values <0·05 were considered statistically significant.

The sample size was not calculated according to a power analysis, due to lack of similar studies. Previous research reporting on the effect of cod proteins in human subjects is based on whole fish^(^^16^^)^ or long-term use of fish protein supplement^(^^17^^,^^29^^)^; thus we did not find any data adequate for making a basis for a power analysis representative for our design. We decided to include forty participants (forty-one were included), a number higher or similar to previously reported in studies on cod protein^(^^16^^,^^17^^,^^29^^)^.

## Results

### Participant characteristics

Overall, forty-one participants completed the trial, of whom twenty-six were female. Mean age was 51 (sd 6) years, range 40–64 years. Mean BMI was 25·2 (sd 3) kg/m^2^. The recorded mean energy intake (2084 (sd 504) kcal/d; 8719 (sd 2109) kJ/d) was lower than the estimated energy need (2605 (sd 392) kcal/d; 10899 (sd 1640) kJ/d) at baseline. The standardised breakfast provided on the study days (500 kcal (2092 kJ)) covered 19·6 (sd 2·9) % of the participants' total energy need. All baseline biochemistry was within the current reference values. Baseline characteristics are presented in Table 3.

**Table 3.** Baseline characteristics of the forty-one participants included in the study at Haukeland University Hospital and Ålesund Hospital between October 2017 and February 2018* (Mean values and standard deviations)

Characteristics	Mean	s**d**
Age (years)	51·0	6·0
BMI (kg/m^2^)	25·2	3·0
Systolic blood pressure (mmHg)	125	18
Diastolic blood pressure (mmHg)	78	11
HbA1c (%)	5·2	0·3
Estimated energy need		
kcal/d	2605	392
kJ/d	10899	1640
Energy intake at baseline		
kcal/d	2084	504
kJ/d	8719	2109
Carbohydrates (g/d)	226·7	68·5
Fat (g/d)	90·2	33·0
Protein (g/d)	92·9	23·6

* Nutritional values are based on mean values from 3-d dietary records.

### Energy intake

Mean energy intake before study day 1 was 2030 (sd 550) kcal/d (8494 (sd 2301) kJ/d). Mean intake before study day 2 was 2110 (sd 534) kcal (8828 (sd 2234) kJ/d). The energy intake did not differ before the two study days (*P* = 0·201).

### Postprandial measurements

Data at each time point are presented in Table 4. In a multivariable, repeated-measures linear mixed-effects regression analysis, no differences were observed between MPH and control for glucose concentration (mean difference: −0·04 (95 % CI –0·17, 0·09) mmol/l; *P* = 0·573). Mean fasting glucose levels were numerically equal on both study days (5·1 (sd 0·4) mmol/l; *P* > 0·999). The peak in glucose concentration (*C*_max_) occurred 20 min after the meal for both MPH and control and was numerically higher after MPH than after the control drink (7·6 (sd 1·8) *v.* 7·4 (sd 1·5) mmol/l, respectively; *P* = 0·997). The AUC was compared for the nine glucose measurements. The AUC for the glucose concentration was numerically equal between MPH (1078 (95 % CI 956·0, 1199·0) mmol/l × min) and control (1068 (95 % CI 944·8, 1190·0) mmol/l × min; *P* = 0·910).

**Table 4.** Descriptive statistics* of the forty-one participants included in a study at Haukeland University Hospital and Ålesund Hospital between October 2017 and February 2018, evaluating the effect of marine protein hydrolysate (MPH) from Atlantic cod (*Gadus morhua*) on postprandial glucose metabolism measured by serum glucose, insulin and glucagon-like peptide 1 (GLP-1) during exposure to MPH and control (casein) drinks (Mean values and standard deviations)

		MPH					Control				
Outcome	Time	Mean	sd	Log mean	sd	GM	Mean	sd	Log mean	sd	GM
Glucose (mmol/l)	Baseline	5·1	0·4				5·1	0·4			
	0 min	6·5	0·9				6·7	0·8			
	20 min	7·6	1·6				7·4	1·5			
	40 min	6·5	1·8				6·2	1·9			
	60 min	5·4	1·4				5·4	1·6			
	80 min	4·9	1·2				5·1	1·4			
	100 min	4·6	1·1				4·7	1·2			
	120 min	4·5	1·1				4·4	1·0			
	180 min	4·4	0·6				4·3	0·6			
Insulin (mIU/l)†	Baseline	6·4	5·8	1·6	0·7	4·9	6·1	5·6	1·5	0·7	4·6
	0 min	33·8	34·3	3·2	0·9	23·8	34·9	30·4	3·3	0·7	27·5
	20 min	69·6	52·7	4·0	0·6	57·1	68·0	47·7	4·0	0·6	56·3
	40 min	64·8	51·1	4·0	0·6	52·0	70·3	53·6	4·0	0·7	55·1
	60 min	57·2	46·2	3·8	0·6	45·7	61·4	49·0	3·9	0·6	49·3
	80 min	42·9	33·9	3·6	0·6	35·0	51·7	47·3	3·7	0·7	39·7
	100 min	36·4	39·3	3·3	0·7	26·9	40·8	42·0	3·4	0·7	31·3
	120 min	28·6	31·3	3·0	0·8	20·6	30·1	37·9	3·0	0·8	21·1
	180 min	12·2	17·7	2·1	0·9	8·0	12·4	15·1	2·2	0·8	8·7
GLP-1 (pmol/l)	Baseline	6·2	9·5	1·5	0·6	4·4	6·2	9·5	1·5	0·6	4·4
	0 min	8·1	9·1	1·9	0·6	6·5	8·8	9·9	1·9	0·6	7·0
	20 min	8·0	9·4	1·9	0·5	6·4	7·9	9·3	1·8	0·6	6·2
	40 min	7·2	9·1	1·7	0·6	5·6	7·3	9·0	1·7	0·6	5·7
	80 min	6·9	10·3	1·6	0·6	5·0	7·0	9·6	1·6	0·6	5·2
	180 min	6·8	10·1	1·6	0·7	4·8	6·5	0·8	1·6	0·6	4·8

GM, geometric mean (exp^(log mean)^).

* Log mean and GM are presented for non-normally distributed data (insulin and GLP-1). Glucose values are only presented as means and standard deviations due to approximately normal distribution.

† In a mixed-model linear regression analysis, the insulin levels were significantly lower after intake of MPH than control (*P* = 0·032).

The insulin concentration was significantly lower after MPH compared with control (mean difference between geometric means: 1·067 (95 % CI 1·01, 1·13) mIU/l; *P* = 0·032). Mean fasting insulin levels were numerically higher before MPH (6·4 (sd 5·8) mIU/l) than control (6·1 (sd 5·6) mIU/l; *P* > 0·999), but the insulin concentration peaked at a lower level and at 20 min (69·6 (sd 52·7) mIU/l) after MPH whereas the peak after the control drink was numerically higher and occurred at 40 min (70·3 (sd 53·6) mIU/l). Women had significantly lower insulin concentrations than men (mean difference between geometric means: 0·65 (95 % CI 0·45, 0·93) mIU/l; *P* = 0·020), irrespective of intervention.

**Fig. 3.**
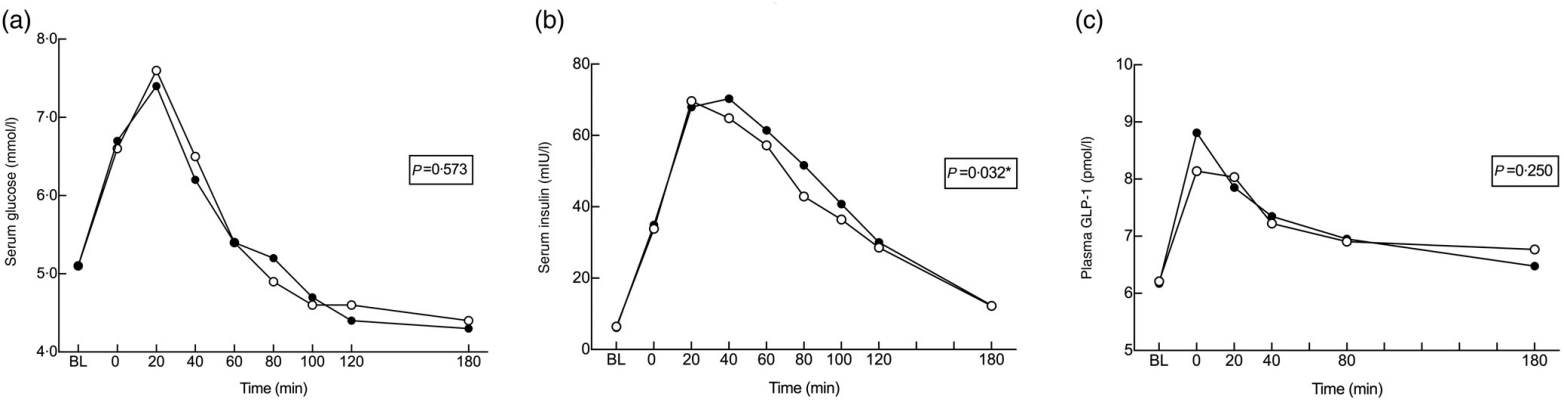
Metabolic response for serum glucose (a), serum insulin (b) and plasma glucagon-like peptide 1 (GLP-1) (c) concentrations after intake of a standardised breakfast meal supplemented with a drink containing either marine protein hydrolysate (MPH; –○–) or control (casein; –●–). Results are presented for forty-one healthy subjects. The study had a cross-over design and all subjects received both treatments in random order. Time point 0 min shows values measured right after the intake of breakfast and test material. Values are means and *P* values are based on a multivariable, repeated-measures linear mixed-effects regression analysis. BL, baseline.

No differences were observed between MPH and control for GLP-1 concentration (mean difference between geometric means: 1·02 (95 % CI 0·99, 1·06) pmol/l; *P* = 0·250). Mean fasting GLP-1 levels were numerically equal on both study days (6·2 (sd 9·4) pmol/l; *P* > 0·999). The peak occurred right after intake of breakfast and test drink (0 min) and was lower after MPH (8·1 (sd 9·1) pmol/l) than after control (8·8 (sd 9·9) pmol/l; *P* = 0·092). Results are presented in Fig. 3.

### Adverse events

No adverse events were reported in the questionnaires or otherwise observed.

## Discussion

The study was designed to investigate the effect of a low dose of MPH on postprandial glucose metabolism in healthy individuals. Our hypothesis was that supplementation with MPH before a meal would beneficially affect the glucose response, insulin and GLP-1 concentration compared with control. We found that a single dose of 20 mg/kg body weight MPH pre-meal supplement significantly lowered the postprandial insulin response. Although we did not observe a reduction in postprandial blood glucose values and GLP-1 concentrations, we postulate that our findings could indicate a potential beneficial effect of MPH in individuals with reduced insulin sensitivity. We hypothesise that MPH may enhance the insulin sensitivity and affect other mechanisms involved in the blood glucose uptake in peripheral tissue. The study participants were healthy individuals with HbA1c levels within the normal range (Table 2), thus one would expect normal blood glucose concentrations after a meal. We speculate that the effect of MPH on postprandial glucose metabolism will be more distinct if further investigated in individuals with the metabolic syndrome or T2DM.

The target of nutritional diabetic and pre-diabetic treatment is to maintain a blood glucose level within the normal range. Several studies have previously shown different sources of protein preload before a meal to reduce the postprandial glycaemic response, both in healthy and diabetic individuals^(^^9^^–^^13^^)^. However, to our knowledge, data on the specific acute effect of a fish protein hydrolysate supplement prior to a meal has previously not been published.

Our finding is consistent with a previous study, showing lower postprandial insulin C-peptide levels after a 7-d intervention with cod^(^^30^^)^. Furthermore, Ouellet *et al*.^(^^16^^)^ have previously demonstrated that a diet rich in cod improved insulin sensitivity in nineteen insulin-resistant individuals, when compared with a diet rich in other animal protein sources. Also, it has previously been demonstrated that cod protein-fed rats, in comparison with casein-fed and soya-fed rats, are protected against the development of insulin resistance and hyperglycaemia induced by diets rich in fat and sucrose^(^^28^^)^. This effect was related to enhanced insulin-stimulated glucose uptake in muscle cells, but not in adipose tissue. It is indicated that amino acids derived from cod protein can increase the insulin-stimulated glucose uptake in muscle cells by acting directly on the glucose transport system^(^^28^^)^. Investigations of the mechanisms promoting this positive effect of amino acids from cod revealed that dietary cod protein restored insulin-induced activation of the phosphatidylinositol 3-kinase (PI3K)/protein kinase B (Akt) pathway and improved translocation of GLUT4 to the T-tubes in skeletal muscle cells^(^^31^^)^. The glucose transporter protein GLUT4 facilitates the uptake of glucose into the cell when expressed at the cell surface, and it has been proposed that a reduced translocation of GLUT4 to the T-tubules leads to the development of insulin resistance^(^^31^^)^. It is proposed that the amino acids derived from cod protein, in comparison with amino acids derived from other protein sources, facilitate a unique pathway leading to the increased expression of GLUT4 in the T-tubules and enhanced insulin sensitivity^(^^31^^)^.

The assumed beneficial effect of the amino acids derived from cod can possibly be linked to the high concentration of branched-chain amino acids (BCAA). It has previously been demonstrated that serum levels of the BCAA leucine, isoleucine and valine, as well as the amino acid lysine, is correlated with the insulin response^(^^32^^)^. The effect has been linked to the increase of hormones such as glucose-dependent insulinotropic polypeptide and GLP-1^(^^33^^)^. Although it is established that the BCAA leucine and isoleucine are the major amino acids affecting blood glucose homeostasis, the effect has not been observed when the amino acid concentration is low^(^^34^^)^. Interestingly, a significant stimulation of glucose uptake in muscle cells by the PI3K/Akt pathway has been observed when the BCAA were administered as dipeptides in low concentrations^(^^35^^)^. The MPH used in our trial have a high concentration of BCAA (Table 1), and analysis of the MPH used in the present study shows that about 10 % of the di- and tripeptide fractions are present as leucine- and isoleucine-containing peptides (data obtained from Firmenich Bjørge Biomarin AS). Even though we did not observe an increase of GLP-1 in relation to the intake of single, low dose of MPH, our findings suggest that low concentrations of MPH may increase insulin sensitivity. The casein used as the control has higher concentrations of BCAA than the MPH, but it differs from MPH regarding the fraction present as di- or tripeptides. The casein used in the study is not a hydrolysate, but present as whole protein, and does not contain either peptides or free amino acids. Thus, we assume that the BCAA-containing peptides present in MPH constitute the unique, bioactive effect even when given in low concentrations. We postulate that this is due to the rapid absorption of intact bioactive leucine- and isoleucine-containing peptides via peptide transporters in the upper jejunum and into the blood. It has been shown that other sources of protein, such as casein and whey, are necessary in much higher doses than MPH to achieve significant alterations in the postprandial blood glucose and insulin response^(^^36^^,^^37^^)^.

One could argue that the control drink should be a true placebo and only contain glucose (maltodextrin), and no protein. However, then it would be possible that the observed effect could simply be due to differences in energy and N content. To avoid this, the control drink contained casein, a protein shown not to affect blood glucose response and insulin sensitivity when given in low concentrations^(^^28^^,^^38^^,^^39^^)^, to facilitate an isoenergetic and isonitrogenic placebo material. Both the MPH and casein control drinks contained an equal amount of protein, in total on average 1·6 g. This amount is negligible compared with the total protein content of the breakfast meal provided (12·5 g protein); thus the effect of MPH can be attributed to the content of bioactive peptides and not the protein *per se*. In a clinical study comprising of 120, slightly overweight (BMI between 25 and 30 kg/m^2^) male and female subjects, Nobile *et al*.^(^^40^^)^ showed that oral doses of 1·4 and 2·8 g MPH from the codfish species blue whiting (*Micromesistius poutassou*) taken daily for 90 d increased the blood concentrations of both cholecystokinin and GLP-1. Further, body weight composition was improved in favour of reduced body fat mass. Daily doses higher than 1·4 g did not give any further effects, demonstrating that MPH may show bioactivity in humans when taken orally in the range of 15–20 mg per kg body weight.

Previous studies have investigated the long-term effect of fish protein intake in overweight, obese and/or diabetic individuals. Improvement in postprandial glucose regulation after intake of 750 g fatty fish/week (for 8 weeks) in overweight/obese adults has been demonstrated, but this effect was not observed after intake of lean fish^(^^15^^)^. Similar findings have been reported in T2DM patients; Zhu *et al*.^(^^41^^)^ demonstrated that treatment with a fish protein hydrolysate improved glucose and lipid metabolism, resulting in reduced fasting blood glucose, insulin and HbA1c, compared with placebo. Vikøren *et al*.^(^^17^^)^ were the first to investigate the specific effect of a fish protein supplement on postprandial blood glucose. They found that low doses of a fish protein supplement from cod (3 and 6 g) for 4 weeks resulted in lower levels of fasting and postprandial glucose, including lower AUC for glucose when compared with placebo, in thirty-four overweight individuals. Another recent study found that supplementation with cod protein for 8 weeks in forty-two overweight and obese individuals had a beneficial effect on postprandial concentration of serum NEFA, but no effect was observed in postprandial glucose or insulin concentration compared with control^(^^29^^)^. The most obvious difference when comparing these studies with our study design is that they evaluated the long-term effect of fish intake/fish protein supplement in overweight and obese patients, while we were interested in the acute effect of a fish protein hydrolysate after a meal in healthy individuals.

There are elements with our design that may have affected the outcome. Previous studies in human subjects have investigated the effect of fish or fish protein supplementation over a longer period of time. Thus, it will be interesting to investigate a potential effect using different doses given over a period of time. Furthermore, we investigated the effect of MPH in healthy individuals, assumed to have a normal glucose response. Our findings indicate that further research should aim to include individuals with hyperglycaemia or abnormal postprandial glucose control. The participants in this study might have been too healthy to find a meaningful effect. The significant lower insulin concentration observed after intake of MPH could be important in patients with reduced insulin sensitivity, thus should be further investigated in a group of patients with the metabolic syndrome and/or T2DM. It has to be considered that 1 week of wash-out for the use of *n*-3 supplements before inclusion might not have been enough; thus the short wash-out period may be regarded as a limitation to our design.

Most previous studies have been performed in rodents, and few data exist on the specific effect of MPH supplement in human individuals. The effect of a low dose of MPH on the postprandial glycaemic response has previously just been hypothesised, and our study is the first to investigate this possible association. Thus, this double-blinded cross-over trial investigating the effect of MPH supplement in human subjects can be regarded valuable for future studies. We suggest that the potential effect of MPH should be investigated over a longer period, with higher doses in patients with impaired glycaemic response and reduced insulin sensitivity.

In conclusion, our findings demonstrated that a single dose of MPH before a breakfast meal reduced postprandial insulin concentration without affecting blood glucose response or GLP-1 levels when compared with control (casein), in healthy, middle-aged individuals. The mechanism for this effect is unknown, and further studies are warranted in target groups with abnormal glucose metabolism.
