# Effect of a cod protein hydrolysate on postprandial glucose metabolism in healthy subjects: a double-blind cross-over trial — CORRIGENDUM

**DOI:** 10.1017/jns.2018.30

**Published:** 2019-01-18

**Authors:** Hanna Fjeldheim Dale, Caroline Jensen, Trygve Hausken, Einar Lied, Jan Gunnar Hatlebakk, Ingeborg Brønstad, Dag Arne Lihaug Hoff, Gülen Arslan Lied

**Affiliations:** 1 Department of Clinical Medicine, Centre for Nutrition, University of Bergen, Bergen, Norway; 2 Division of Gastroenterology, Department of Medicine, Haukeland University Hospital, Bergen, Norway; 3 National Centre of Functional Gastrointestinal Disorders, Haukeland University Hospital, Bergen, Norway; 4 Firmenich Bjørge Biomarin AS, Ellingsøy, Ålesund, Norway; 5 Department of Clinical Medicine, University of Bergen, Bergen, Norway; 6 National Centre for Ultrasound in Gastroenterology, Haukeland University Hospital, Bergen, Norway; 7 Division of Gastroenterology, Department of Medicine, Ålesund Hospital, Møre & Romsdal Hospital Trust, Ålesund, Norway; 8 Department of Clinical and Molecular Medicine, Faculty of Medicine and Health Sciences, Norwegian University of Science and Technology, Trondheim, Norway

**Original text and correction:**

**ORIGINAL TEXT (page 3, Subjects and methods)**

Fig. 1. Flowchart depicting the inclusion process for the study evaluating the effect of a marine protein hydrolysate (MPH) from Atlantic cod (*Gadus morhua*) on postprandial glucose metabolism in healthy individuals aged 40–65 years. Participants were recruited through advertisements on the Internet and posters at Haukeland University Hospital and Ålesund Hospital between October 2017 and February 2018.

Fig. 2. Study protocol for the evaluation of the effect of a marine protein hydrolysate (MPH) from Atlantic cod (*Gadus morhua*) on postprandial glucose metabolism. We included forty-one healthy subjects (age range 40–64 years).

**CORRECTION**

Fig. 1. Study protocol for the evaluation of the effect of a marine protein hydrolysate (MPH) from Atlantic cod (*Gadus morhua*) on postprandial glucose metabolism. We included forty-one healthy subjects (age range 40–64 years).

Fig. 2. Flowchart depicting the inclusion process for the study evaluating the effect of a marine protein hydrolysate (MPH) from Atlantic cod (*Gadus morhua*) on postprandial glucose metabolism in healthy individuals aged 40–65 years. Participants were recruited through advertisements on the Internet and posters at Haukeland University Hospital and Ålesund Hospital between October 2017 and February 2018.

**ORIGINAL TEXT (page 8, Acknowledgements)**

E. L. is Professor Emeritus at the University of Bergen, Bergen, Norway and the managing director of Science of Firmenich Bjørge Biomarin AS, Ellingsøy, Ålesund, Norway. The other authors declare no conflict of interest.

**CORRECTION**

E. L. is Professor Emeritus at the University of Bergen, Bergen, Norway, and former Scientific Advisor of Firmenich Bjørge Biomarin AS, Ellingsøy, Ålesund, Norway, where he holds a royalty agreement. The other authors declare no conflict of interest.
